# Monkeypox (Mpox): a comprehensive review of epidemiology, therapeutic advances, and public health implications

**DOI:** 10.1186/s41043-026-01323-9

**Published:** 2026-04-20

**Authors:** Amit Verma, Sandeep Sharma, Gopal Lal Khatik, Ashish Suttee, Dinesh Kumar, Neeraj Choudhary, Suresh Babu Kondaveeti

**Affiliations:** 1 Mahatma Jyotiba Phule Rohilkhand University, Bareilly, U.P India; 2 Faculty of Allied Medical Sciences, Lovely Professional University, Punjab, India; 3 National Institute of Pharmaceutical Education and Research, Raebareli, India; 4 School of Pharmaceutical Sciences, LPU, Phagwara, Punjab India; 5 GNA School of Pharmacy, GNA University, Phagwara, Punjab India; 6 Symbiosis Medical college for Women & Symbiosis University Hospital and Research Centre, Symbiosis International (Deemed University), Pune, 412115 India

**Keywords:** Monkeypox virus (MPXV), Mpox, Orthopoxvirus, Emerging zoonoses, Disease outbreaks, Epidemiology, Transmission, Diagnostics, Vaccines, Therapeutics, Public health preparedness

## Abstract

**Background:**

Monkeypox, a zoonotic viral disease caused by the monkeypox virus (MPXV), was first identified in captive monkeys in 1958 and in humans in 1970. Although historically limited to Central and West Africa, the unprecedented 2022–2025 multi-country outbreaks exposed its ability for sustained human-to-human transmission, raising urgent global health concerns.

**Objectives:**

This review aims to provide a comprehensive overview of the epidemiology, genomic structure, evolutionary patterns, transmission routes, clinical features, and treatment options related to MPXV.

**Methods:**

Evidence was collected through systematic searches of established scientific databases (PubMed, Scopus, Web of Science, ScienceDirect, Cochrane Library, and Google Scholar) using specific keywords (monkeypox, MPXV, outbreak, transmission, diagnosis, therapeutics, vaccination, public health). Data were carefully analyzed to compare historical and current outbreaks, evaluate diagnostic methods, explore developments in therapeutics and vaccines, and assess public health responses. The review highlights advances in molecular diagnostics (PCR-based platforms, CRISPR-integrated assays, and point-of-care systems), emerging therapeutic options (tecovirimat, brincidofovir, cidofovir), and novel vaccine strategies (MVA-BN/JYNNEOS and ACAM2000). However, barriers such as asymptomatic infections, under-resourced healthcare infrastructure in endemic areas, viral genomic plasticity, and inequitable vaccine access continue to limit effective containment.

**Conclusions:**

By integrating clinical, molecular, and epidemiological perspectives, this review underscores the urgent need for global surveillance networks, targeted vaccination policies, and host-pathogen-driven therapeutic innovations. Addressing these priorities is critical to mitigating future outbreaks and establishing long-term resilience against MPXV and related orthopoxviruses.

## Introduction

The recent multi-country outbreaks of mpox, caused by the monkeypox virus (MPXV), have transformed this formerly neglected zoonotic disease into a global public health priority. Humans are naturally inclined to stay close to the environment and the different life forms in it. Humans, animals, and the environment play a significant role in the transmission of diseases within and between species. Infections and diseases that are transmitted from vertebrate animals to humans and vice versa are termed zoonotic diseases or zoonosis. Zoonosis is a Greek word in which ‘Zoon’ means animals and ‘nosos’ means illness. Zoonotic diseases are categorised according to the types of pathogens. They can be bacterial zoonoses (such as tuberculosis, anthrax, etc.), viral zoonoses (swine flu, monkeypox, rabies, etc.), mycotic zoonoses (ringworm, blastomycosis, etc.), and protozoal zoonoses (toxoplasmosis, leishmaniasis, etc.). Monkeypox is a zoonotic disease caused by the MPXV belonging to the family *Poxviridae* [1, 2]. Monkeypox is a rare viral disease originating from African regions sharing 90% genomic similarity with the smallpox virus, i.e., Variola virus [3]. The disease is not new; it was first detected the first time in the monkeys (*Macaca cynomolgus*) of Singapore origin in 1958, and the virus was isolated at the State Serum Institute in Copenhagen. Its occurrence is not limited to animals, as the first human case was observed in a child in 1970 in the region of Zaire [4]. The symptoms associated with the monkeypox virus (MPXV) are milder than those associated with smallpox disease, due to which it was mistaken earlier for smallpox disease.

This disease became concerning when cases started rising in different regions of the world. Between 2022 and 31 March 2025, a total of 137,892 confirmed cases of mpox have been reported globally, from 127 countries and territories. The Region of the Americas (49.7%) contributes the largest proportion of cases, followed by the African (23.4%) and European (21.4%) Regions. In the Region of the Americas, a cumulative total of 68,553 confirmed cases of mpox, including 151 deaths, were reported in 31 countries and territories between 2022 and 2025. In 2025, a total of eight countries (Argentina, Brazil, Canada, Chile, Costa Rica, Mexico, Paraguay, and the United States) have reported mpox cases, with no recorded deaths to date [5]. Most of the patients suffering from MPXV had a travel history to countries such as North America, the Middle East, and Europe, from where they became infected and further transmitted this disease to another person. Most of the infected persons were men aged between 20 and 49 years, and some of them were homosexuals. The virus spreads from person to person through respiratory droplets, body fluids, or contact with infected lesions [6]. A recent study suggests that MPXV can enter the central nervous system (CNS) through neuroinvasive mechanisms, which are governed by the olfactory epithelium and involve infected monocytes or macrophages, potentially leading to subsequent neurological problems [7].

This comprehensive review synthesizes the current knowledge on MPXV, focusing on its evolving epidemiology, genomic characteristics, transmission dynamics, clinical management, and the public health challenges that remain for effective global control.

## Methods

The research is a narrative review, but with a systematic search of the literature. Using electronic databases, such as PubMed, Scopus, Web of Science, ScienceDirect, database inception to March 2026, the keywords identified as monkeypox, mpox, MPXV, epidemiology, transmission, diagnosis, therapeutics, vaccination, and public health were used to find relevant articles. Peer-reviewed original research articles, reviews, and case reports about human mpox were used as inclusion criteria. Duplicate records, non-English publications, and commentaries and editorials were not included. Recent epidemiological information was also accessed based on the reports by international organizations, including the WHO, CDC, and PAHO. Relevant data on epidemiology, genomics, transmission, diagnosis, therapeutics, and public health response were extracted and synthesized in a narrative fashion to help identify the notable developments and trends.

## Epidemiological trends (historical to 2025)

###  The first outbreak in 1958

In 1958, two pox-like viruses emerged in monkeys in Denmark. Their symptoms were similar yet somewhat distinct from one another, but a lack of proper knowledge led to the misinterpretation of the information later. It was found that 20% of the monkeys were affected by the first outbreak, and 30% of them were affected by the second outbreak. Later, for further research, samples were collected from the affected lesions of the monkeys’ dermal portion. Based on the laboratory results, it was concluded that the virus is similar to the pox virus and was therefore included in the family as the “monkeypox virus” [14]. From clinical observations, it was found that the dermal lesions were pruritic and exhibited umbilication, with a higher incidence of lesions in the palm and sole areas. The lesions were superficial, as no organs were found to be affected in the autopsy reports, and no casualties occurred. In another study, the euthanized monkeys indicated the presence of MPXVs in their kidneys and showed asymptomatic infections [15].

### The first human case, 1970

Following the emergence of the disease in 1958, several outbreaks occurred, but they were often mistaken for smallpox. The first case was diagnosed in a 9month old boy when the program for smallpox eradication surged in the Democratic Republic of the Congo (DRC) in 1970 [16]. The symptoms began with fever, followed by superficial rashes, and a sample of superficial crusts was found to be positive for MPXV clade I. Further cases were reported in different regions of the Congo Basin, including the Central African Republic, DRC, and Cameroon [17, 18]. Later, the WHO initiated a continuous surveillance mode to monitor this virus closely. The data included the records of 282 patients diagnosed with MPXV between 1980 and 1985; 85% of the cases occurred among children in the 0–10 year age group. The fatality rate was 11%, affecting mostly the age group of 0–4 years [19].

###  The emergence of cases in 2003

In the midwestern United States, the incidence of cases increased again in May and June 2003. The outbreak of the disease occurred due to the import of ill Prairie dogs from West Africa to the United States. In 2003, the first case was reported in a three year old girl who was hospitalised due to fever and cellulitis after being bitten by a Prairie Dog. The dog later died due to illness with lymphadenopathy and skin lesions. The enlarged submandibular lymph node was submitted to the laboratory for bacterial culture in Marshfield Laboratories. Reports revealed the contamination of Acinetobacter species, and this event was considered an isolated case. The Wisconsin Division of Public Health (DPH) oversees all reports and documents cases. The Milwaukee Health Department reported the second case to the DPH about the illness of a meat inspector who was involved in the distribution of exotic animals. A Prairie dog bit the inspector, and 3–4 days post-bite, he developed symptoms such as fever, lesions, and chills. Due to the worsened conditions, he was hospitalised and later diagnosed with the disease. An epidemiological connection was established between these two cases, and it was observed that more cases arose due to their connection with previous cases, as they were either involved in the trade of prairie dogs or purchased them. The PCR reports of different patients confirmed the presence of MPXV DNA [20]. The mother of the first patient and their dog also showed symptoms and tested positive for MPXV. An alarming condition emerged in several regions of Illinois, Wisconsin, and Indiana, resulting in a large outbreak with 72 cases, of which some were confirmed, and a few were suspected [21].

### Reemergence in 2017, Nigeria cases

Following the incident in the United States, a surge in cases was observed in various states of Nigeria. The Nigeria Centre for Disease Control (NCDC) notified suspected patients of monkeypox in September 2022. The numbers were huge, so without delay, they were admitted to the Niger Delta University Teaching Hospital in Bayelsa State, Nigeria, and underwent isolation, contact tracing, laboratory testing, and pre-treatment immediately. The first admitted patient was an 11year old boy with symptoms of high fever for 11 days, rashes, sore throat, and headache. He also experienced skin eruptions with umbilication, and rashes were abundant on the trunk, palms, soles of the feet, and face. In addition to superficial lesions, the patient also developed ulceration in the oral and nasal mucosa, as well as lymphadenopathy. The five family members also developed the same symptoms due to contact transmission. Upon tracing the source, it was found that they had a history of contact with the neighbour’s monkey; however, it was not confirmed whether the monkey was infected or not [22, 23]. A total of 38 suspected cases were identified, of which 18 were confirmed by laboratory testing, 3 were suspected, and the remaining 17 cases were found to be improbable to fit in the category of ‘Human Monkeypox’. The major clinical symptoms observed in the patients were fever, rashes, lymphadenopathy, headache, sore throat, itching, and genital ulcers; some also developed symptoms of diarrhoea and scrotal swelling [24].

### Monkeypox outbreak 2022

In 2022, monkeypox spread in several countries outside endemic areas. The first outbreak of human cases in 2022 occurred in Europe and North America. This was caused by the clade of West Africa’s MPXV. The person who travelled to Nigeria from the United States showed the symptoms of MPXV, and after diagnosis, was found positive. In September 2022, the number reached its peak, with a total of 31,800 cases, of which 31,425 were from countries that had not previously experienced the disease. The USA, Spain, and Brazil were the leading countries with the highest number of cases. These unexpected data raised concerns among scientists in different countries [25]. This time, the condition differed from previous reports, as the cases were more numerous and geographically distant from each other, with no details of contact tracing [26]. According to the reports of the Centers for Disease Control and Prevention (CDC), updated on December 28, 2022, for United States cases, the total number of cases was 29,792, with 20 casualties [27].

### Recent updates, 2023

**Fig. 1:**
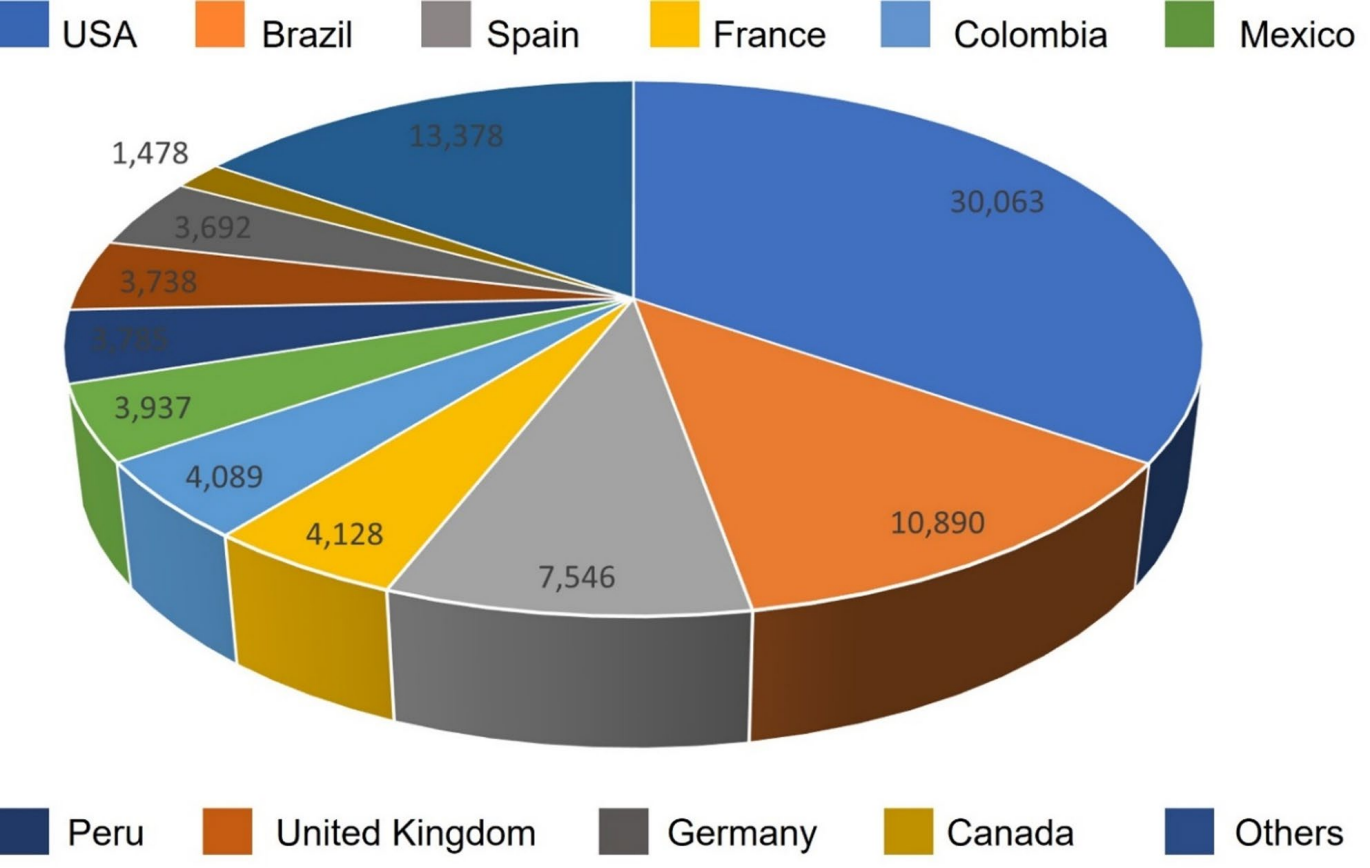
Cumulative number of monkeypox cases worldwide until March 2023

Between January 2022 and March 2023, a total of 86,724 laboratory-confirmed cases of monkeypox were reported from 110 countries (Table 1). A high number of cases (73,346 cases; 84.6% of the world’s cases) originated from 10 main countries: the United States, Brazil, Spain, France, Colombia, Mexico, Peru, the United Kingdom, Germany, and Canada (Fig. 1).

**Table 1:** Number of confirmed monkeypox cases and deaths by the WHO from January 2022 to March 2023

**WHO geographical region**	**Confirmed cases**	**Deaths**
WHO Region	58991	86
European Region	25852	6
African Region	1454	18
Western Pacific Region	301	0
Eastern Mediterranean Region	83	1
South‒East Asia Region	43	1
**Total**	**86724**	**112**

**Fig. 2:**
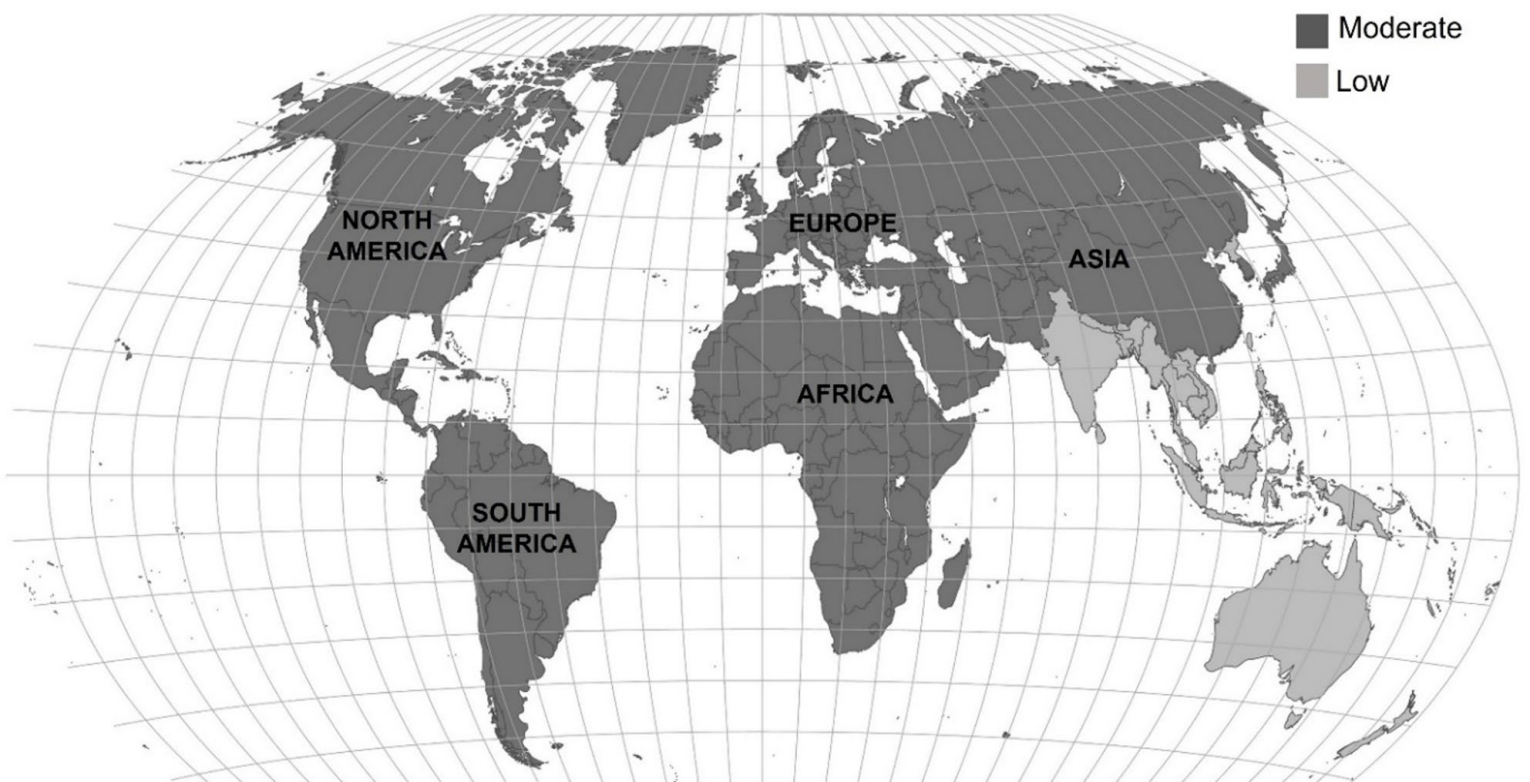
Global regions with a moderate or low risk of monkey pox according to thebe transferred from one animal to another or from animals to humans through close contact, or through the as it can infect both animals and humans

Based on recent updates in March 2023, regions in Africa, the eastern Mediterranean, Europe, and America are at moderate risk, while regions in Southeast Asia and the western Pacific are at low risk for monkeypox incidence (Fig. 2) [28–34].

### Monkeypox outbreak 2024

The global mpox outbreak, which began in May 2022, has resulted in 137,892 confirmed cases across 127 countries by March 2025, primarily caused by the West African clade (clade IIb) [82, 83]. In the United States, a total of 34,730 confirmed cases and 63 deaths were reported during the 2022–2023 outbreak [84]. Concurrently, the Democratic Republic of the Congo experienced a severe clade I outbreak, with over 22,000 suspected cases and 1,200 deaths reported between 2023 and March 2024 [85, 86]. Children accounted for the majority of fatalities in the DRC outbreak [84].

As of 8 March 2024, a total of 94,766 confirmed cases and 182 deaths have occurred in 117 countries [17–19].

The primary animal reservoir for MPXV is unknown, according to reports from the CDC. Small animals, however, can maintain a suitable atmosphere for the growth of the virus in West and Central Africa. Prairie dogs, squirrels, shinchillas, giant-pouched rats, marmots, and groundhogs are the types of rodents that can be reservoirs for MPXV. In the case of non-human primates, monkeys and apes can also be infected. The virus can be transferred from one animal to another or from animals to humans through close contact or through trapping, hunting, and processing of infected parts or body fluids [35]. Till now, three clades of MPXV were the clade of Congo Basin (clade I) and the clades of West Africa (clade IIa and IIb). Clade I contains the genome sequence of the Congo Basin, while clade IIa and IIb are genetically very similar and show 95% nucleotide sequence identity to clade I [36]. Most commonly, clade IIa of MPXV is transmitted from animal to human, while clade IIb transmission occurs from human to human [37]. The disease was endemic to West and Central Africa, but the emergence of cases in the non-endemic regions is surprising and a matter of concern. The primary source of MPXV for these areas is unknown, and the cases showed no contact history with endemic areas. The only hypothesis made was that it may be a zoonotic spill over that is transferred from one animal to another or from animal to human and further from human-to-human contact, either through single or multiple importations of the virus, with or without showing any clinical symptoms during the transmission [38].

## Genome and structure of MPV

**Fig. 3:**
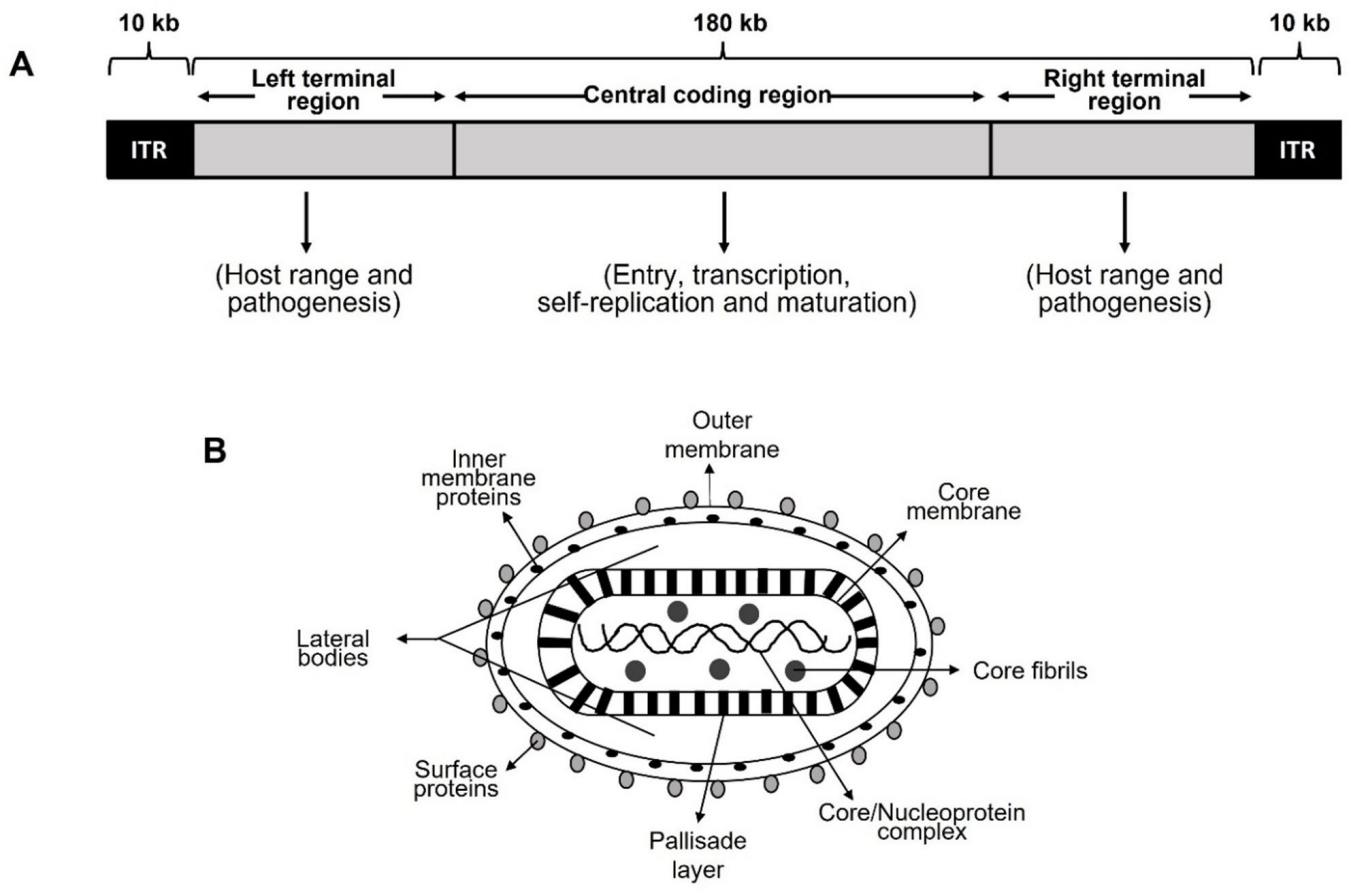
Genome (**A**) and structure (**B**) of monkey pox virus

The genome of the monkeypox virus is linear, with a size of approximately 200 kb (196,858 base pairs), and contains 190 ORFs. The central coding region is conserved and encodes housekeeping genes involved in virus entry, self-replication, transcription, and maturation.^8^ The central region of a genome is flanked by terminal regions that are responsible for the pathogenesis of the virus. Inverted terminal repeats (ITRs) of 10 kb are present at each end of a genome (Fig. 3A) [8–10].

**Fig. 4:**
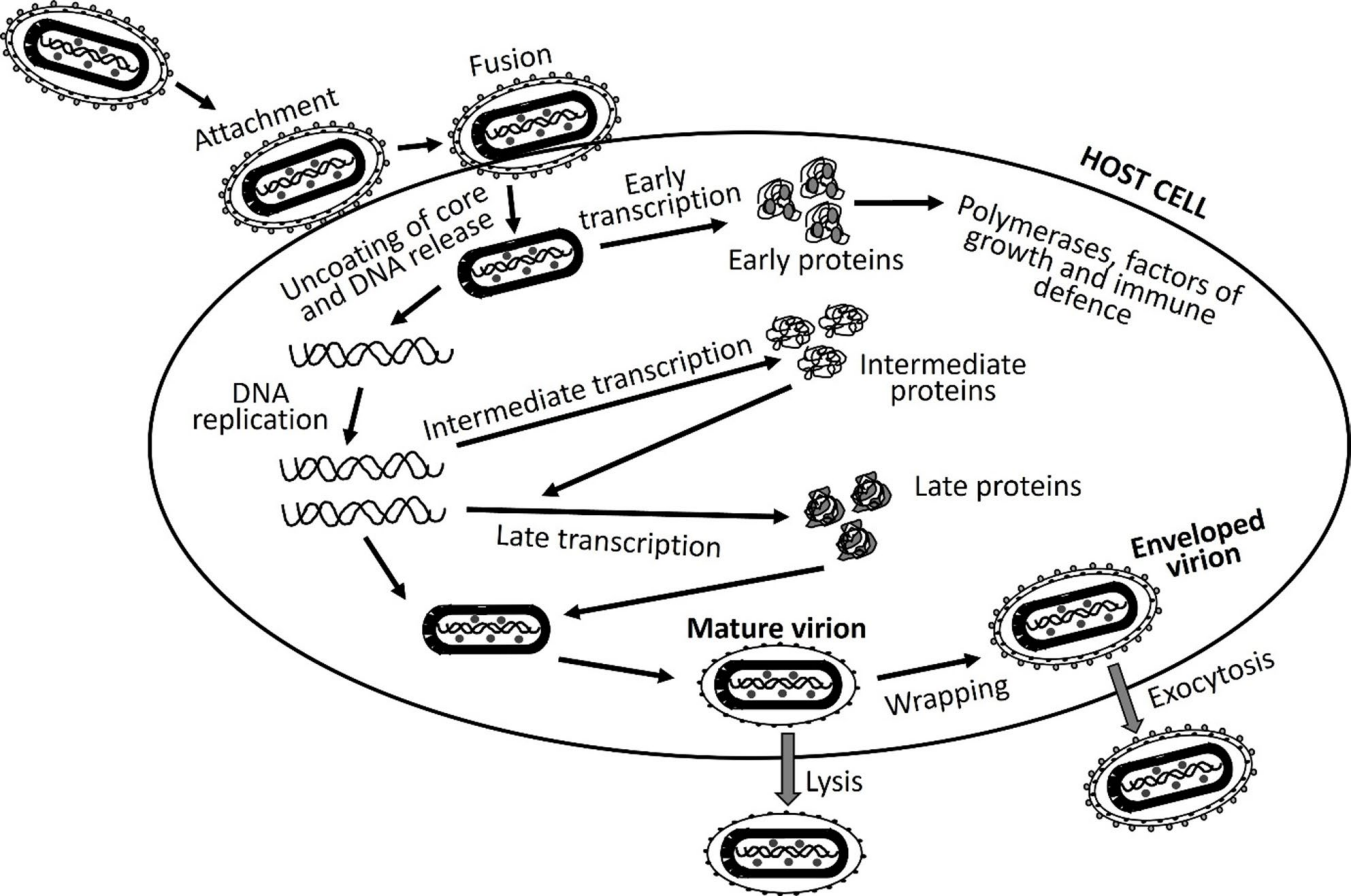
An overview of the life cycle of the monkeypox virus. To infect a person, a virus enters the cytoplasm of a host cell following viral attachment and fusion with the host cell. Afterwards, the virus releases its core into the cell cytoplasm and starts replication. Viral particles are assembled as a mature virion (MV). MV can wrap an additional envelope in the form of enveloped virion (EV) and release from the cell via lysis (for MV) or exocytosis (for EV) [53]

**Fig. 5:**
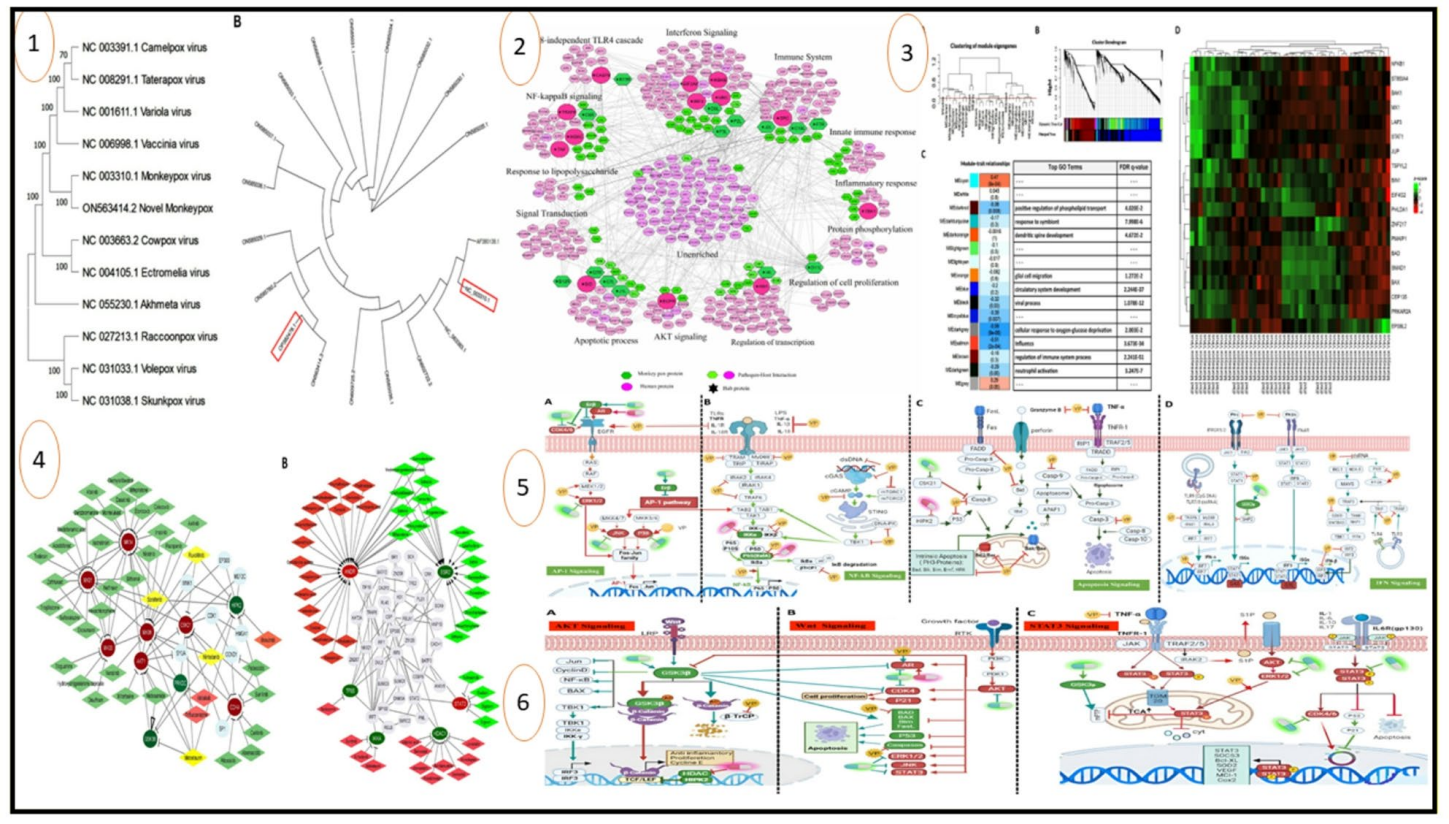
Integrated systems biology and therapeutic insights into Mpox pathogenesis. (**1**) *Phylogenetic analysis of orthopoxviruses* showing Mpox clustering as a distinct monophyletic clade closely related to vaccinia and variola strains (**2**) *Mpox–human protein interaction network* highlighting dense viral-host interactions, with hub proteins (black stars) driving key immune and signaling processes such as NF-κB, AKT, apoptosis, and interferon responses (**3**) *Weighted gene co-expression network analysis (WGCNA)* identifying 16 gene modules; heatmap of 19 shared genes between HPI and WGCNA modules reveals antiviral and proviral clusters linked to immunity, circulatory/metabolic stress, and neutrophil activation (**4**) *Drug repurposing network analysis* using Steiner tree modeling of kinases and transcription factors, mapping FDA-approved drugs targeting proviral (red) and antiviral (green) gene pathways, with dual-targeting drugs in yellow (**5**) *Cell signaling regulation by Mpox* demonstrates proviral activation of AP-1 signaling via MAPK/ERK, suppression of NF-κB pathways, inhibition of both extrinsic and intrinsic apoptosis, and disruption of interferon (IFN) signaling (**6**) *Mpox-driven manipulation of host signaling pathways* showing activation of AKT signaling via mTORC2 and PDK1, stabilization of β-catenin through Wnt signaling, and non-canonical STAT3 activation by D3R-mediated phosphorylation, revealing therapeutic intervention points. Copyright Imani et al.., 2024 [87].

The structure of the monkeypox virus is brick-shaped, large (length: 220 to 450 nm and width: 140 to 260 nm), and complex [11, 12]. It has four major components: the outer membrane, outer lipoprotein envelope, core, and lateral bodies [13]. The surface proteins are present on the outer surface. The outer membrane holds the core, lateral bodies, and palisade layer. The core is a central region that comprises ds DNA and core fibrils (Fig. 3B). 

### Effect of evolution on MPXV adaptation, transmission, and pathogenesis

Characterization of the evolution patterns of coding regions of the viral genome can help us to understand the mechanisms underlying the emergence and viral pathogenicity. It has been reported that MPVX evolution is not driven by intra-species homologous recombination [55]; however, higher recombination of MPXVgp090 (an RNA polymerase subunit) and MPXV182 (a surface glycoprotein) was observed in some poxviruses [56, 57]. For the detection of proteins involved in the adaptation process via natural selection, dN/dS (the ratio of substitution rates) was measured, and it was observed that ≈ 64% of coding genes have an average dN/dS < 0.5. These signs indicate that negative selection is the main reason behind evolution in MPXV [57]. MPXV genes, which are associated with virus-host interactions including MPXVgp015 (kelch-like protein) [58], MPXVgp016 (IL-1 receptor antagonist) [59], MPXVgp024 (α-amanatin target protein) [60], MPXVgp033 (caspase-9 inhibitorshowed positive selection at the whole genome level with high dN/dS (> 1.5) [69]. Ten other genes also showed positive selection, and a few involved in host range determination like ankyrin genes (MPXVgp004, MPXVgp010, MPXVgp012, MPXVgp014, MPXVgp178, and MPXVgp188) [62–64]; MPXVgp004 (EEV maturation protein), MPXVgp138 (a component of IMV surface tubules), MPXVgp098 (belongs to subunit of mRNA capping enzyme) and MPXVgp191 (a chemokine binding protein) [70]. These results revealed that genes under positive selection at the codon level probably contribute to transmission and pathogenesis of MPXV.

Till now, three clades of MPXV are known, and phylogenetic analysis suggested that genetic drifts, duplication of genes, horizontal gene transfer, or lineage actively participate in the evolutionary history of MPXV [64, 65]. It was found that 80% of positive amino acid (AA) substitutions emerged many years ago. Three positive AA substitutions T/A426V, A423D, and S105L in MPXVgp010, MPXVgp012, and MPXVgp191, respectively, were identified in 2019 or 2022, representing that such mutations are crucial for viral adaptation to humans [71].

### Life cycle

MPXV contains DNA as nucleic acid, and its replication occurs in the cell cytoplasm. The entry of poxvirus into the host cell can be completed in three steps: (i) the attachment of virus to the host cell, (ii) fusion of virion with the membrane of host cell and (iii) entry of viral core into the cytoplasm of host cell via endocytosis and release of core in cytoplasm (figure-4). For viral attachment, several glycosaminoglycans such as heparin sulfates, chondroitin, and laminin etc. [41]. contribute; afterwards, virion binds to the membrane with the help of viral proteins like D8L [42, 43], A27 [44, 45], A34R [46, 47], A26L [48] and H3L [49, 50] and unites with the host cell by releasing it’s core in the cytoplasm of cell. The enzymes and factors contained in the core initiate transcription via DNA-dependent RNA polymerase, which is followed by translation of early, intermediate, and late proteins. The cooperation of host-derived transcription factors is required for the viral transcription of intermediate and late stages [51–53]. Inside the cell cytoplasm, most of the virions are enclosed within a protein matrix called intracellular mature virion (IMV), while some of them obtain a second envelope to attach to the host cell membrane and are known as intracellular enveloped virion (IEV). These cell surface-associated enveloped virions (CEVs) cause virus transmission from one cell to another, and some extracellular enveloped virions (EEVs) govern systemic transmission.

### Host pathogen interaction and invasion mechanism of the human monkeypox virus

In case of infection transmission through animals, the virus is found in the excreted matter and secretions of the animals and in their skin lesions. From animals, the virus is able to reach different organisms or humans either by inhalation or direct contact with their infected parts or body fluids. It can also be transmitted from one person to another through sexual contact. The person who got infected by the MPXV showed lower mild symptoms compared to other poxvirus infections. The signs start with flu-like symptoms such as headache, fatigue, chills, body aches, vomiting, cough, and sore throat. Conditions such as lymphadenopathy and skin lesions on the palms and soles arise but resolve within 14–21 days. Some patients show conjunctivitis, corneal ulceration, and keratitis, and the respiratory system may become infected and can develop bronchopneumonia [54, 61]. The incubation period for the MPXV is 7–14 days, but it can go up to 21 days [27].

General and severe symptoms for monkeypox and its similarity with symptoms associated with smallpox infection are mentioned in Table 2.

**Table 2:** Symptoms of monkeypox virus infection and similarities with smallpox virus infection

**Symptoms/incubation period**	**Features**	**References**
General symptoms of monkeypox	Back pain, Fever, Headache, Lymphadenopathy, Myalgia, Skin lesions	[54, 67]
Severe symptoms of monkeypox	Conjunctivitis. In immunocompromised patients, splenomegaly and hepatomegaly	[68]
Symptoms are common in both monkeypox and smallpox infection	Fever, Muscular Pain, Skin lesions (Lymphadenopathy is absent in smallpox infection)	[68]
Incubation period of Monkeypox and smallpox	6-13 days	[68]

There are three routes through which the virus enters the human body, i.e., the nasopharynx, oropharynx, and the intradermal routes. After reaching the host, it replicates at the inoculation sites and targets the lymph nodes. Later, through the blood, the virus infects other organs.

The brick-shaped or oval-shaped virus is surrounded by a lipoprotein membrane. The life cycle of the linear double-stranded DNA of MPXV is processed in the cytoplasm of the cell. The membrane containing viral DNA is attached to the host cell and then enters it either by macropinocytosis or by fusion [27]. The cell envelope is of two types, i.e., mature virions and enveloped virions. After entering the cell, the cell envelope is removed, and viral DNA is released into the cytoplasm. This viral DNA goes into the process of early transcription, mRNA is formed, and if the viral DNA follows the process of DNA replication, late transcription, and late translation, then multiple copies of the mature virion and enveloped virion are formed. They are released outside the cell and infect others. The genome of viral DNA encodes proteins that are necessary for replication, translation, transcription, and virion assembly. Once the host cell is infected with the virus, it results in two contagious viruses: one is a mature virion, and the other is an enveloped virion [27, 68].

## Case studies

The case study on Mpox combines genomic, proteomic, and therapeutic perspectives. It begins with phylogenetic analysis that places Mpox within the orthopoxvirus family as a monophyletic group alongside vaccinia and variola. The global phylogeny emphasizes close genetic similarities between the 2022 outbreak strain and previously known variants. Moving to host–pathogen dynamics, the Mpox-human protein interaction network shows a dense interaction between viral and host proteins, with central hub proteins driving vital biological processes, highlighting their potential as therapeutic targets. Complementing this, WGCNA-based co-expression network reconstruction identifies 16 gene modules, with heatmaps displaying 19 common genes that distinguish antiviral from proviral responses. These genes relate to immune regulation, circulatory and metabolic stress, neutrophil activation, and shared viral pathways like influenza, defining Mpox’s immunopathogenic footprint. Translating these findings into intervention strategies, drug repurposing analysis using Steiner tree modeling uncovers disrupted kinase and transcription factor pathways, mapping antiviral and proviral gene networks, and highlighting FDA-approved drugs with potential to target these processes. Mechanistically, cell signalling modulation shows Mpox’s ability to activate AP-1 signalling via MAPK/ERK, inhibit NF-κB activity, suppress both extrinsic and intrinsic apoptosis, and interfere with IFN responses at multiple levels—all of which facilitate immune evasion. Finally, Mpox’s manipulation of host proviral signaling demonstrates its capacity to stabilize β-catenin through Wnt activation, enhance AKT phosphorylation via mTORC2 and PDK1, and activate STAT3 through D3R-mediated non-canonical phosphorylation. These processes support viral persistence but also indicate potential targets for therapeutic intervention, as shown in figure-5 [87].

**Fig. 6:**
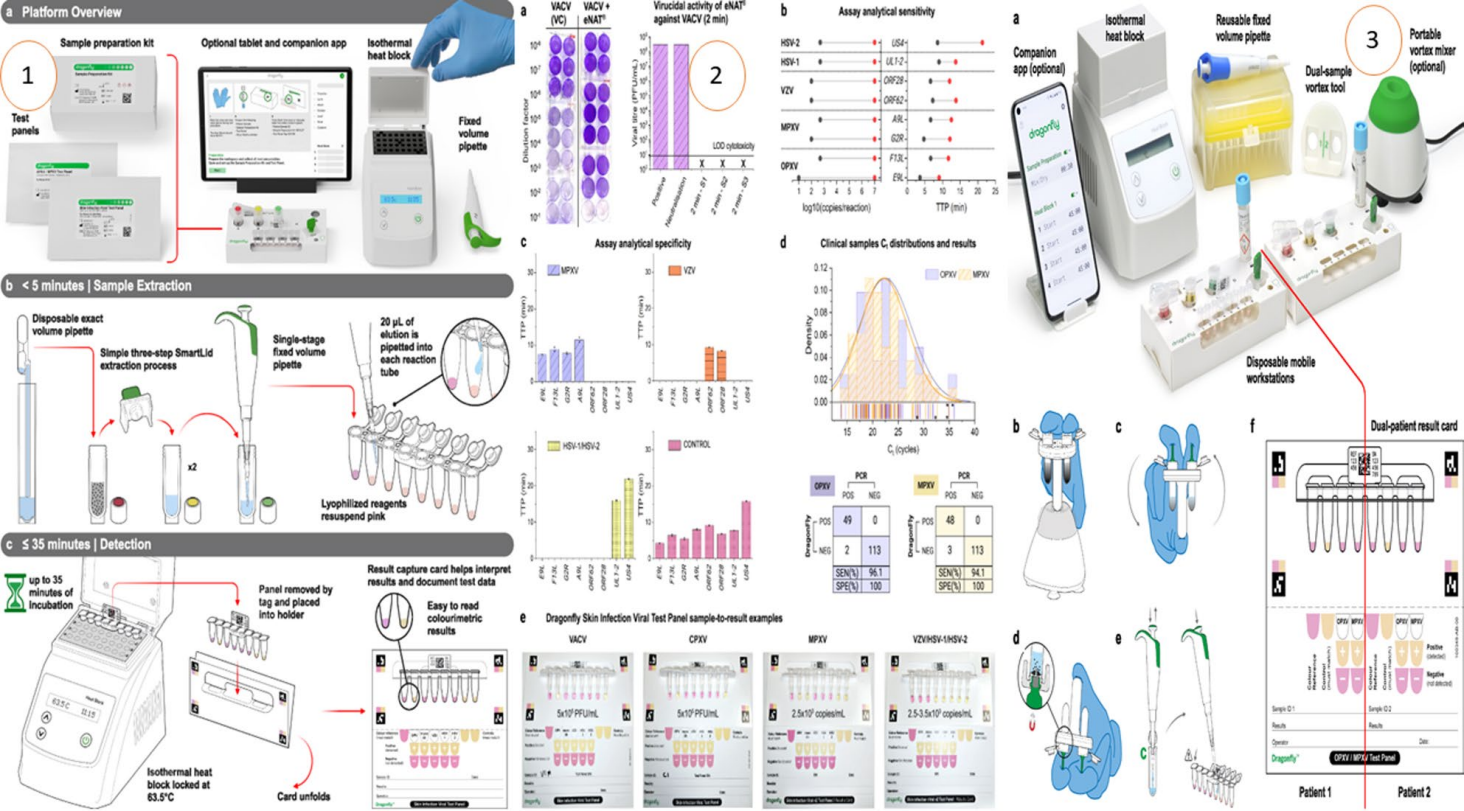
Portable point-of-care molecular diagnostic platform for Mpox detection. (**1**) Overview of the platform components, including sample preparation kit, extraction tools, isothermal heat block, lyophilized reagents, and companion app (**2**) Analytical validation of the assay: (**a**) virucidal activity of eNAT^®^ against vaccinia virus, (**b**) assay sensitivity with synthetic DNA, (**c**) specificity against MPXV, VZV, and HSV-1/2, and (**d**) distribution of qPCR Ct values from clinical samples with corresponding confusion matrices; (**e**) representative colourimetric results for different viral targets (**3**) Dual-patient configuration of the system: (**a**) complete mobile testing kit, (**b**) dual-sample vortex tool, (**c**–**d**) simultaneous magnetic bead collection, (**e**) sequential test panel loading, and (**f**) example of dual-patient result card with valid negative results. Copyright Cavuto et al., 2025 [88].

This case study demonstrates the development and validation of a portable molecular diagnostic platform designed for the rapid point-of-care detection of Mpox and other orthopoxviruses. The platform consists of a sample preparation kit, disposable pipettes, an isothermal heat block, lyophilized reagents, and an optional tablet-based companion app for workflow guidance and results visualization. Sample extraction is achieved in under five minutes using a simple three-step SmartLid process, after which the nucleic acids are transferred to the reaction panel. Detection takes place within 35 min, with colourimetric readouts enabling easy interpretation of results on a capture card. To ensure biosafety, the eNAT^®^ solution was tested for virucidal activity, demonstrating an 8-log reduction in vaccinia virus within two minutes, as confirmed by plaque assays. Analytical validation of the LAMP assay showed high sensitivity across a wide dynamic range, detecting synthetic DNA copies per reaction, and high specificity with no cross-reactivity to HSV-1, HSV-2, or VZV. In clinical validation, the distribution of Ct values from qPCR confirmed reliable detection of OPXV and MPXV, with confusion matrices demonstrating high sensitivity and specificity compared to standard PCR tests. A dual-patient workflow was also introduced, featuring a reusable vortex tool, concurrent bead-based extraction, and sequential panel loading to prevent cross-contamination. Results for both patients are summarised in a dual-patient result card, ensuring efficiency in outbreak situations, as shown in Fig. 6 [88]. 

**Fig. 7:**
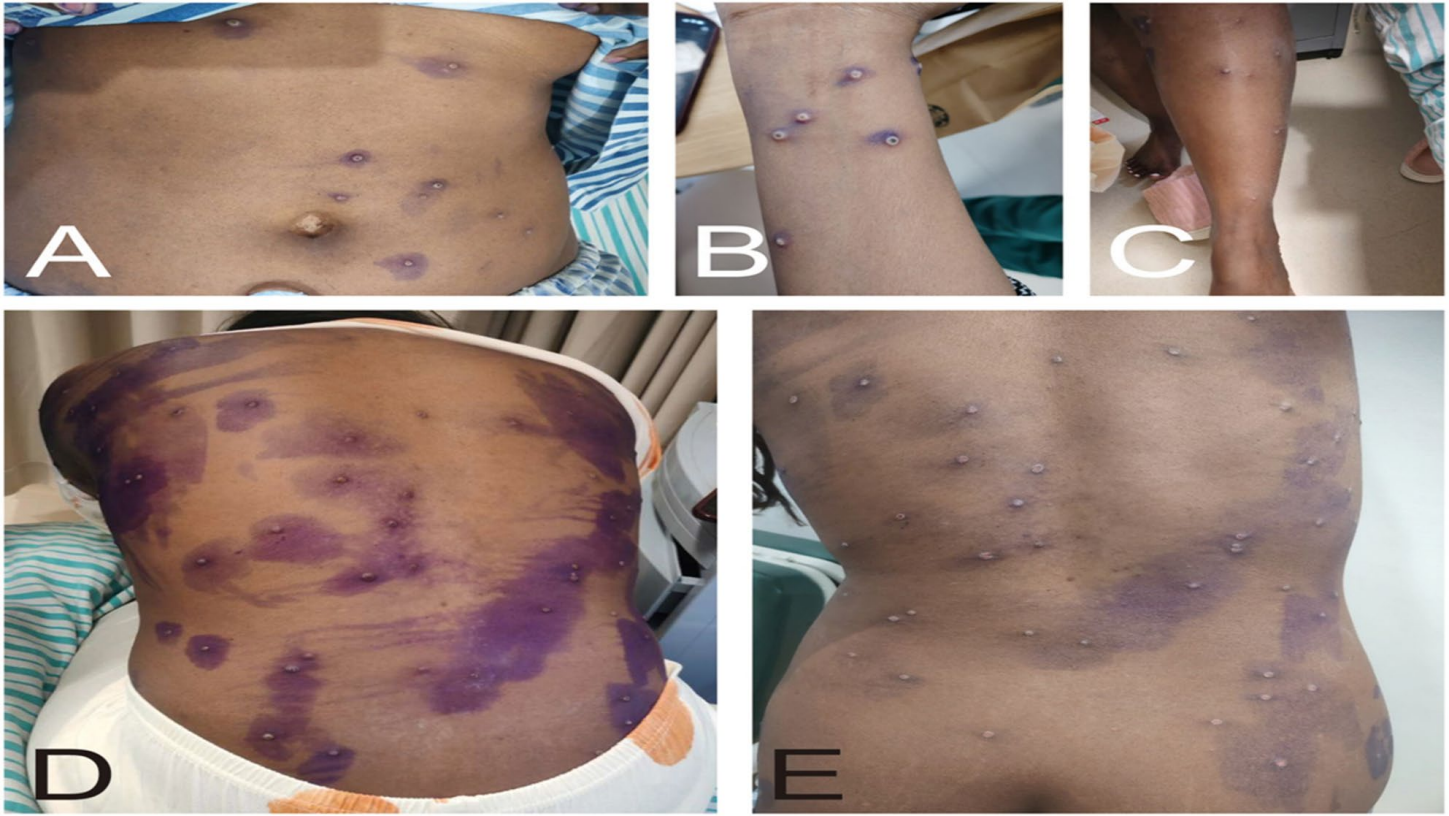
Clinical presentation of Mpox clade Ib infection in a 28year old woman. (**A**) Pustular lesions on the abdomen (December 30, 2024) (**B**) Lesions on the arm (December 30, 2024) (**C**) Lesions on the lower leg (December 30, 2024) (**D**) Disseminated pustules with scabbing on the back (December 31, 2024) (**E**) Shedding scabs on the back during recovery (January 3, 2025). Copyright W. Pan, R. Ge, G. Zhu et al.2025 [89]

**Fig. 8:**
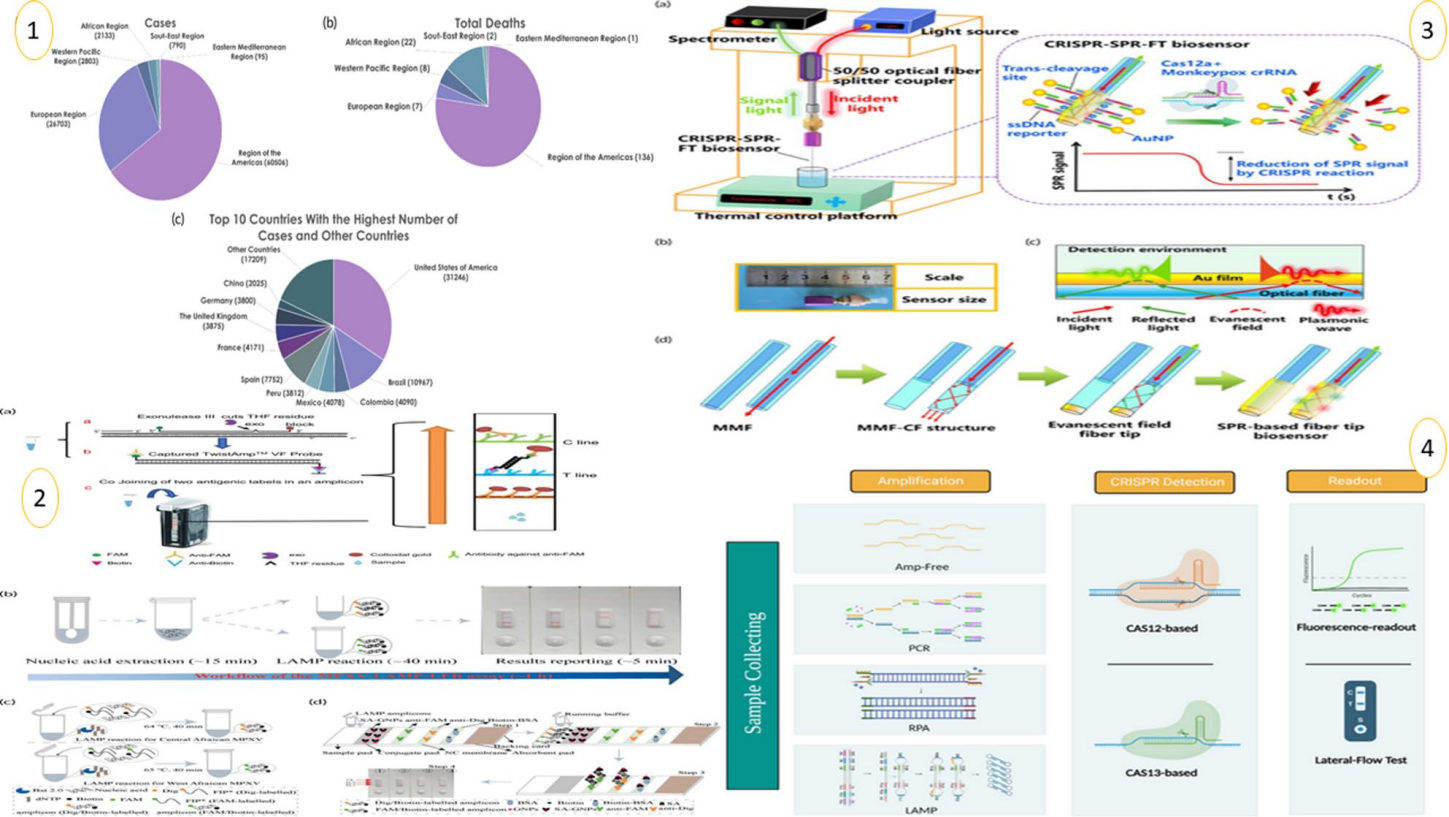
Global epidemiology and emerging diagnostic platforms for Mpox detection. (**1**) Regional and country-level distribution of Mpox cases and deaths, showing the Americas and Europe as the most affected regions (**2**) Development of lateral flow assays integrated with nucleic acid amplification (LAMP) and enzymatic probe cleavage for rapid detection (**3**) CRISPR-SPR-FT biosensor platform utilizing Cas12a-mediated cleavage and fiber-tip surface plasmon resonance for ultrasensitive detection of Mpox DNA (**4**) Overview of diagnostic workflows integrating amplification (PCR, RPA, LAMP), CRISPR-based detection (Cas12 and Cas13), and readouts (fluorescence and lateral-flow assays), demonstrating versatile strategies for point-of-care testing. Copyright Atceken et al., 2025 [90]. Infected person to Healthy person

A 28year old South African woman living in eastern China developed Mpox caused by clade Ib, marking the first such deviation from the previously predominant clade IIb in the region. Her illness began on December 21, 2024, with malaise and myalgia, progressing to pustular eruptions on December 30 that involved the trunk, limbs, buttocks, and face but spared the genital and oral regions. PCR confirmed diagnosis with Ct ~ 22, validated by the Chinese CDC on January 2, 2025. During hospitalization, lesions evolved to scabbing by January 1 and resolved by January 13, with full recovery and no complications. Epidemiology linked infection to sexual contact with a male partner from the Democratic Republic of Congo, supported by positive environmental samples, though no secondary transmission occurred among more than 100 contacts. Clinically, her presentation contrasted with clade IIb Mpox, which usually involves fever, prominent lymphadenopathy, and genital lesions. The absence of fever and genital involvement, along with generalized pustular eruptions, highlighted distinct clinical features of clade Ib. This case underscores the need for early molecular testing, active surveillance, and thorough contact tracing, especially among high-risk groups (women, children, immunocompromised) in vaccine-limited regions. It also suggests possible differences in epidemiology and transmission pathways of clade Ib Mpox, including heterosexual and potential vertical transmission, shown in figure-7 [89].

This figure presents the dual perspective of the global epidemiological burden of Mpox and the advances in diagnostic technologies that enable rapid detection and control. The epidemiological analysis shows that the Americas and Europe recorded the highest case numbers, followed by Africa and the Western Pacific, with overall mortality remaining low but unevenly distributed across regions. Countries such as the United States, China, Germany, the UK, France, and Brazil reported the largest caseloads, reflecting Mpox’s widespread international transmission. To address the diagnostic challenges, several innovative tools have been developed, including lateral flow assays combined with nucleic acid amplification (LAMP) and enzymatic probe cleavage, providing rapid on-site results within an hour. Further advances include a CRISPR-SPR-FT biosensor platform, which uses Cas12a-mediated nucleic acid cleavage and fiber-tip surface plasmon resonance for highly sensitive, real-time detection of Mpox DNA in portable formats. Complementary approaches integrating amplification methods (PCR, RPA, LAMP), CRISPR-based detection (Cas12 and Cas13), and readout systems (fluorescence or lateral flow assays) highlight the versatility of point-of-care platforms. Collectively, these technologies, when aligned with global surveillance data, emphasize the importance of coupling epidemiological monitoring with rapid, field-deployable diagnostic systems to strengthen outbreak response and mitigate the spread of mpox shown in figure-8 [90].

## Transmission factors

- High risk factors: Direct physical contact via sexual or intimate contact with infected persons. Men who have sex with multiple partners and persons who frequently have sex with multiple partners are at higher risk.
- Low risk factors: Contact with contaminated material (e.g., bleeding) and virus spread via fluid droplets from blisters/mouth/nose.

**Fig. 9:**
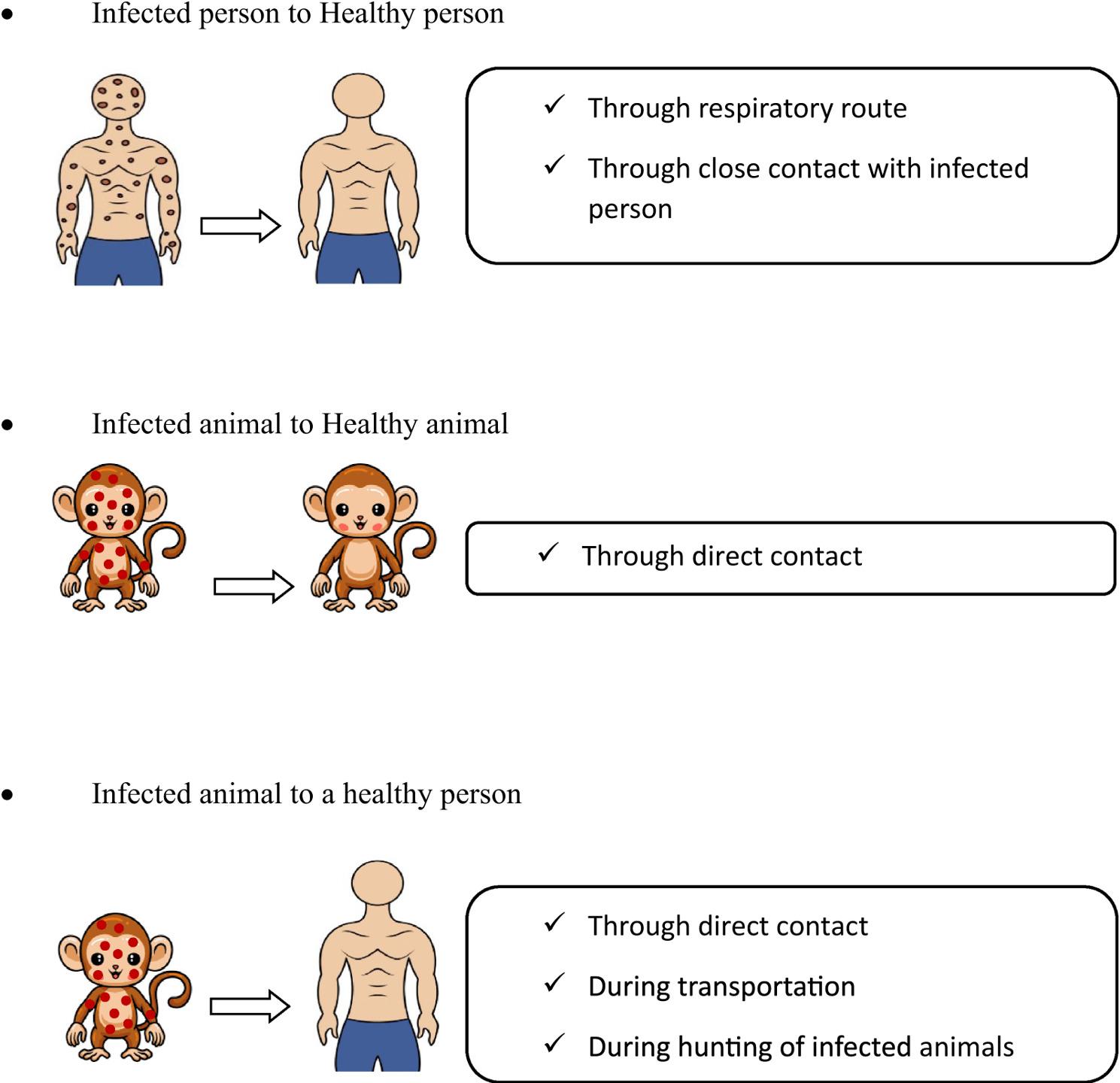
Monkeypox virus transmission route between human and non-human primates

The routes of transmission of MPXV between humans and animals are summarized in Fig. 9.

## Diagnosis

Several laboratory methods, such as conventional polymerase chain reaction (PCR), nested multiplex PCR, Real-time quantitative PCR (RT-qPCR), digital PCR, Robotic process automation (RPA), gene sequencing (GeneXpert), loop-mediated isothermal amplification (LAMP), and microarray-based assay, were approached to diagnose the infection of MPXV [39]. Among these methods, the detection of unique viral DNA sequences by conventional PCR or RT-qPCR alone or together with gene sequencing is recommended by the World Health Organization (WHO). If PCR is not available, then serological tests can be performed for the detection of infected patients. Higher levels of anti-*Orthopoxvirus* IgM & IgG antibodies in the blood sample of the patient (within 5–8 days after rash appearance) confirm the monkeypox infection. The suggested specimens can be collected directly from skin rashes, fluid, or crusts by vigorous swabbing. Testing can be performed on oropharyngeal, anal, or rectal swabs when skin lesions are absent [40].

## Potential therapeutic strategies against the monkeypox virus

Monkeypox is a self-limiting disease, but sometimes it may become fatal and can also cause death if not treated well. The specific treatment, especially for MPXV, is not clear, but due to its similarity with smallpox, the antiviral drugs employed for smallpox treatment, such as brincidofovir and tecovirimat, find clinical applications. Brincidofovir (lipid conjugate of cidofovir) has activity against double-stranded viral DNA and good oral bioavailability. Tecovirimat was also tested on the squirrel model, and all animals survived the lethal challenge when tecovirimat was given to them for 0–3 days against MPXV. However, both drugs require further human trials to validate their effectiveness [81]. Drugs available for the treatment of monkeypox infection are shown in Table 3.

**Table 3:** Details of drugs used for the treatment of monkeypox virus-infected patients

**Drug**	**Year of approval**	**Mechanism**	**Side effects**	**Dosage form**	**References**
Cidofovir	1996	It acts on DNA synthesized by viral DNA polymerase and inhibits it	Nausea, vomiting, headache, renal failure	Solution	[73]
Tecovirimat	2018	It acts on VP37 viral protein and inhibits its attachment with cellular TIP47 and Rab9 GTPase	Abdominal pain, vomiting, nausea, headache	Capsules	[72, 73]
Brincidofovir	2021	Inhibits viral DNA synthesis	Diarrhea, abdominal pain, vomiting	Tablets Suspension	[66, 67]
Trifluridine		Inhibiting thymidylate synthetase, replication of the virus gets disrupted	Minimal side-effects	Ointment Topical Antibiotic drops	[74]

### Prevention measures

To control the transmission of MPXV, we need to follow the instructions as mentioned below:-.

- Avoiding contact with infected persons, animals (usually rodents and primates), and contaminated materials.
- Using PPE (personal protective equipment) while caring for or dealing with virus-infected persons.
- Practicing hand hygiene (washing hands with soap/disinfectant), safe sex (use of condoms and dental dams), and thoroughly cooking animal meat.
- Vaccination with JYNNEOS vaccine (an approved primary vaccine for monkeypox) and ACAM200.

### Vaccine for monkeypox virus

Vaccination is an important way to limit the spread of monkeypox infection. The CDC recommends vaccination for people against MPXV who fall under the following categories:

- If the person comes in contact with the suspected patients.
- If the person had sex with the partner in the past 2 weeks, and the partner was diagnosed with monkeypox infection.
- If the person is working in the laboratory who deals with the orthopoxviruses.
- If the person is gay or bisexual and has more than one male sex partner who is diagnosed with a sexually transmitted disease.

MPXV and smallpox virus belong to the same viral family, so the CDC recommends the JYENNEOS vaccine against them. Including this vaccine, which works for smallpox infection, also shows protective action against MPXV. Modified Vaccinia Ankara (Jyenneos) and ACAM200 are the two existing vaccines against both diseases. Jyenneos^®^ is recommended in Australia for safety purposes, as it is easy to administer, and the Australian government made the supply of Jyenneos^®^ at a faster pace under section 18 A of the Therapeutic Goods Act 1989. The clinical features of vaccines are discussed in Table 4 [74–79].

**Table 4:** Details of vaccines used to treat monkeypox virus-infected patients

**Vaccine**	**Route of administration**	**Doses**	**Booster dose (if required)**	**Who can be vaccinated**	**Side effects**
Modified vaccinia ankara (Jyenneos)	Subcutaneous route	Twice with a gap of 28 days	Every 2 years	Children Pregnant women Immunocompromised person	Mild pain and fatigue
ACAM2000	With the help of a bifurcated needle, multiple punctures.	Once	Every 3 years	Only for children	Mild; rarely severe (post-vaccination encephalitis)

A recent study reported the potential of multi-epitope vaccine constructs MPXV-2 and MPXV-5 against MPXV with the help of immunoinformatics. Both constructs can elicit the immune responses very efficiently against MPXV and could be the possible vaccine candidates in the future [78].

### Major obstacles in research progress

MPXV showed evolutionary changes over time. The study and surveillance of the disease sometimes become quite difficult for various reasons. The two different clades of MPXV showed some differences in their symptoms and behaviour.

The major obstacles in the surveillance of this virus are as follows:

- Asymptomatic conditions arise that are difficult to diagnose over time. These asymptomatic patients transmitted the disease from one to another, and the primary source became undetectable.
- Monkeypox disease was previously misdiagnosed as smallpox, and the poor laboratory conditions of developing countries detected it later.
- Endemic areas for this disease have unskilled staff and workers and inappropriate knowledge about the genetics of the disease, making it difficult to treat on time [77, 78].

There is a requirement for a strong network of health officials for contact tracing of the emerging source of MPXV. Many of the experts’ hypotheses conclude that the emerging and re-emerging cases of monkeypox in both endemic and nonendemic areas were due to factors such as climatic changes, highly mobile populations, armed conflict in these areas and low immunity in society. The major obstacle in research progress has been the undetected form of the virus. Its occurrence in nonendemic areas may be due to its transfer from one person to another or from one country to another through the mobile population in an undetected form for weeks or months [79].

The disease was first reported in 1958 and, as of September 2022, had spread to 106 countries. The genomic data were collected from different sources for the detection of mutations in the viral genome. DNA polymerase of MPXV, specifically F8L and VACV E9 (the catalytic subunit of DNA polymerase), was isolated to identify the mutation. In the catalytic subunit, VACV E9, three residues, S951, A684, and A498, showed an increased frequency of mutation. Ten mutations were identified in the virus replication complex; two were in the catalytic subunit of F8L, and two were in the processivity factor G9R in the 2022 sequences [80]. Mutating genes in the virus cause problems in research factors and can cause more harm to the individual.

## Future perspectives

The resurgence and global spread of monkeypox highlight the urgent need for long-term strategies to manage zoonotic viral diseases. Future work should prioritize continuous genomic and evolutionary surveillance to monitor mutations that may enhance viral transmissibility or virulence. At the same time, investment in innovative diagnostics and therapeutics is critical,particularly the development of affordable, portable, and rapid tests alongside next-generation antivirals and monoclonal antibody therapies that can be deployed in both well-resourced and resource-limited regions. Equitable access to vaccines such as MVA-BN (JYNNEOS) and ACAM2000, as well as the advancement of novel multi-epitope vaccine candidates, will be essential to ensure effective global protection. Strengthening vaccination campaigns with strong community engagement, digital surveillance tools, and cross-border collaboration can improve preparedness and response. Above all, adopting a One Health approach,recognizing the close links between humans, animals, and the environment,will be central to reducing the risk of future outbreaks. By combining scientific innovation with public health equity, the global community can shift from a reactive posture to a sustainable and resilient model for combating monkeypox and other emerging pathogens.

## Discussion and limitations

According to this review, there has been a significant epidemiological change of mpox since 2022 due to clade IIb and the perpetuation of human-to-human transmission in non-endemic areas, whereas clade I persistently causes more severe illness in endemic ones. These results suggest a dual pattern of transmission with clinical and health implications in the region. Increased response to outbreaks has been achieved through the use of improved diagnostics (PCR, CRISPR-based assays), improved therapeutics (tecovirimat, brincidofovir), and improved vaccines (JYNNEOS, ACAM2000). Nonetheless, unidentified cases, unusual manifestations, and new modes of transmission pose a challenge to the conventional surveillance and control measures. Nevertheless, even with improvements, major challenges, such as inadequate diagnostic capacity, unequal access to vaccines, inadequate genomic surveillance, and lack of healthcare infrastructure, especially in endemic areas, still persist. Viral development and clade variability also make it hard to control. It presents a more holistic view of the topic and connects epidemiology with molecular processes and translational interventions compared with current reports (e.g., WHO, CDC). On the whole, mpox is a chronic health issue faced all over the world, and it needs a coordinated, equity-based, and One Health intervention. This review is limited in a number of ways. Being a narrative review, it is prone to possible selection bias. The use of published data creates potential reporting bias and change in the quality of data. Some of the findings might be temporary due to the speed at which the outbreak is changing. Also, the non-English studies can be excluded, which can restrict the world representation.

## Conclusion

This review provides a summary of the existing knowledge on mpox, its epidemiology, viral genomics, modes of transmission, clinical manifestations, and diagnostic and therapeutic interventions. Molecular diagnostics advances over the recent years, including PCR-based tests, CRISPR-based tests, and point-of-care platforms, have enhanced the process of detecting and tracking mpox outbreaks. Likewise, antiviral drugs (tecovirimat and brincidofovir) and vaccines (JYNNEOS and ACAM2000) give valuable disease control and prevention opportunities. Nevertheless, there are a number of challenges. The ongoing change of the virus, viral clade disparities, lack of diagnostic capability in certain areas, and disparate access to vaccines and health services remain relevant to positive disease control. Thus, the world needs to be monitored better, people have to be more prepared for health issues, and access to diagnostics and vaccines should be enhanced. A One Health approach, where human beings, animals, and the environment are linked, will be needed to prevent future outbreaks and to control the long-term spread of mpox.

## Data Availability

No datasets were generated or analysed during the current study.

## Declarations

### Clinical trials

This study is not a Clinical Trials and therefore did not require trial registration. 
